# Effect of neoadjuvant treatment with anastrozole on tumour histology in postmenopausal women with large operable breast cancer

**DOI:** 10.1038/sj.bjc.6600435

**Published:** 2002-08-01

**Authors:** T J Anderson, J M Dixon, M Stuart, T Sahmoud, W R Miller

**Affiliations:** Department of Pathology, Western General Hospital, Edinburgh EH4 2XU, UK; Breast Unit, Western General Hospital, Edinburgh EH4 2XU, UK; AstraZeneca, Alderley Park, Cheshire SK10 4TG, UK

**Keywords:** neoadjuvant, anastrozole, postmenopausal women, histology, pathology

## Abstract

Anastrozole is an orally active, non-steroidal aromatase inhibitor which appears effective as neoadjuvant treatment of breast cancer. Histological changes have been evaluated in biopsies from large, oestrogen-receptor rich, operable breast tumours in postmenopausal women following 12 weeks of neoadjuvant anastrozole treatment (1 mg (*n*=12) or 10 mg (*n*=11)). Of the 23 patients, 18 had a clinical response following treatment. Compared with pre-treatment biopsies anastrozole-treated specimens displayed decreased cellularity and/or increased fibrosis in 15 tumours; changes in gland formation, nuclear pleomorphism, or mitoses, in 12 cases; and a reduction in Mib1 score in all tumours. Marked changes in apoptotic scores were seen following treatment but the direction of effect was inconsistent. In all 17 tumours which were positive for progesterone receptors before therapy, treatment was associated with reduced staining for progesterone receptors. There was no consistent effect of treatment on oestrogen-receptor expression. It is concluded that neoadjuvant anastrozole treatment in this patient group has marked effects on tumour histopathology but these do not always correlate with clinical response.

*British Journal of Cancer* (2002) **87**, 334–338. doi:10.1038/sj.bjc.6600435 www.bjcancer.com

© 2002 Cancer Research UK

## 

Although surgery is the most important mode of primary treatment for most breast cancers, neoadjuvant treatment can be a useful part of the clinical management of many patients. By giving appropriate drugs before surgery, tumours may shrink such that less extensive surgery is needed and, in particular, allow breast-conserving surgery in patients who might otherwise have required a mastectomy before neoadjuvant treatment (Miller *et al*, 1999). Neoadjuvant treatment also provides a valuable opportunity to study the effect of drugs on a variety of biochemical and histological features of the tumour. Whilst chemotherapy is the most frequent therapy used in the neoadjuvant setting, endocrine therapy has also been evaluated in hormone-sensitive, large, operable or locally advanced breast cancer (Anderson *et al*, 1989; Valero *et al*, 1989; Leal da Silva *et al*, 1998).

Anastrozole is a potent, non-steroidal inhibitor of aromatase, the enzyme responsible for catalysing the conversion of androgens to oestrogen. The drug markedly suppresses oestrogen levels in postmenopausal women, in whom peripheral aromatase activity is the main route of oestrogen production (Miller, 1996; Bajetta *et al*, 1999). Clinical trials in postmenopausal women with advanced breast cancer have shown that anastrozole is well tolerated (Buzdar *et al*, 1998). When used as second-line therapy in patients who have progressed on tamoxifen treatment, it is significantly superior to megestrol acetate in terms of overall survival (Buzdar *et al*, 1998). In addition, anastrozole has greater efficacy in terms of prolonged time to progression, compared with tamoxifen, as first-line therapy for patients with advanced breast cancer known to be hormone receptor-positive (Buzdar *et al*, 2000; Nabholtz *et al*, 2000; Bonneterre *et al*, 2001), as well as having tolerability advantages. Thus, anastrozole is now challenging the place of tamoxifen as first-line treatment of advanced breast cancer. Studies have indicated that anastrozole is also effective in the neoadjuvant setting, producing clinically important reductions in tumour volume and mastectomy rates (Dixon *et al*, 2000).

Although it has been established that anastrozole suppresses oestrogen levels within the tumour when used for neoadjuvant treatment (Geisler *et al*, 1999), the precise mechanisms by which this oestrogen deprivation exerts its clinical effect are not well understood. The aims of this study were therefore to determine the effects of 12 weeks' treatment with anastrozole on the morphology, histological grade, proliferative activity, and steroid receptor status of breast cancers, in order to gain a greater understanding of the mode of action of this drug.

## MATERIALS AND METHODS

The study was approved by the appropriate ethics committee, and was conducted in accordance with the ethical principles of the Declaration of Helsinki. All patients gave written informed consent before any trial-related procedures were performed.

### Patients

The study included postmenopausal women with large, operable, oestrogen-receptor (ER)-rich (ER shown on the initial core biopsy to be >80 by histoscore) breast cancer. Postmenopausal status was defined as patients aged 50 years or over who had not menstruated in the past 12 months, or women of any age with follicle-stimulating hormone (FSH) levels >40 IU l^−1^. For inclusion in the study, patients had to have operable breast cancers >3 cm (T_2_ >3 cm, T_3_) or locally advanced breast cancer (T_4b_). Full demographic and baseline tumour characteristics of the patients have been reported previously (Dixon *et al*, 2000) and are summarized in Table 1

**Table 1:**
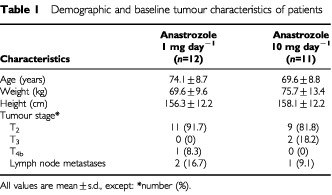
Demographic and baseline tumour characteristics of patients

.

### Study design

The results presented here are secondary outcomes from a previously published single-centre study which was designed primarily to assess the effects of anastrozole on peripheral and intra-tumour aromatase activity (Dixon *et al*, 2000). Patients were randomized in a 1 : 1 ratio to double-blind neoadjuvant treatment with anastrozole 1 or 10 mg (once daily) for 12 weeks. Before anastrozole treatment started, breast tissue was removed by wedge biopsy to determine variables including baseline morphology, histological grade, proliferative activity, and steroidal receptor status. At the end of the 12-week treatment period, tumours were removed by curative-intent surgery. The tumours removed during surgery were analysed to determine the effects of treatment on the above variables.

### Assessments

#### Tumour volume

Our previous experience, based on excision biopsies, has shown that ultrasound measurements are generally more accurate than calliper or mammographic assessments (Forouhi *et al*, 1994). Therefore, tumour volume was assessed pre-treatment and at monthly intervals by ultrasound, as previously described (Dixon *et al*, 2000). Results were calculated as the percentage change in volume between measurements made before treatment and after three months' therapy. In previous publications (Bajetta *et al*, 2000; Zilembo *et al*, 2000) a reduction of >25% in tumour volume is taken as evidence of clinical response (assessments made in accordance with the International Union Against Cancer guidelines). However, in the present study, all tumour volume reductions were greater than 50%.

#### Histopathology

The histopathological features of the tumour removed by surgery at the end of the study were compared with the initial wedge biopsy. Tumour morphology was judged by comparing changes in cellularity and fibrosis. Histology was also scored for grading features according to the method described by Elston and Ellis (1991). This method involves the histological assessment of three components of tumour morphology: tubule formation, nuclear pleomorphism and frequency of mitoses, and an overall tumour grade (1 to 3) is calculated.

#### Cell proliferation – Mib1 staining

Ki67 antigen was assayed, by measuring the binding of a mouse monoclonal antibody, Mib1, to the Ki67 nuclear antigen, using sections taken from a pre-treatment tumour biopsy sample and from a post-treatment surgical specimen. The percentage of cells staining in a minimum of 10 representative high-power fields was used to quantify Mib1 expression (Gerdes *et al*, 1983).

#### Apoptotic index

Apoptotic (cell death) index was measured using the TUNEL immunohistochemical technique (Lebat-Moleur *et al*, 1998). The index was defined as the number of apoptoses per 1000 cells and was derived using methodology originally used to assess mitotic index (Simpson *et al*, 1992).

#### Oestrogen and progesterone receptors

Oestrogen receptor and progesterone receptor (PgR) status were assessed by immunohistochemical techniques after microwave antigen retrieval, using ID5 (Dako) for ER status and PG88 (Biogenix) for PgR status. The results were scored on a scale of 0 to 3 for intensity (with each successive score denoting increasing intensity), and on a score of 0 to 5 for proportion (with the greatest proportion denoted by a score of 5); the values were then summed into a category score with a range of 0 to 8 (Allred *et al*, 1998).

### Statistical methods

The study size was chosen in order to analyse the primary endocrinological endpoints. The statistic analyses for pathology parameters were therefore applied without previous power calculations. Because numbers of patients in each dose group were small and there is no *a priori* reason to believe that pathology responses are dose-related, analyses have been performed combining the dose groups (although for information tables and figures present data separately).

## RESULTS

A total of 12 patients were eligible for analysis in the anastrozole 1 mg group, and a total of 11 patients were available in the anastrozole 10 mg group.

### Histopathological assessments

Marked morphological changes with treatment were evident in the majority of tumours (15 of 23; 65%). These constituted both decreased cellularity and increased fibrosis in 11 cases, and decreased cellularity alone in two; in two tumours there were only microscopic foci of disease after treatment. All these effects were taken as evidence of a pathology response. Additionally, changes in grading characteristics were associated with treatment in 12 of the tumour pairs. Tubular features were increased in five cases, nuclear pleomorphism was decreased in four, and mitotic index was decreased in five cases but increased in one.

The relationship between pathological response and ultrasound volume change is shown in Table 2

**Table 2:**
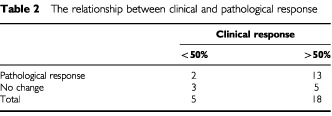
The relationship between clinical and pathological response

. This indicates that pathological changes with treatment may be observed in tumours that have no ultrasound response. Conversely, whilst the majority of tumours with a major reduction in ultrasound volume also show pathological response, five tumours (of 18) were without evidence of pathological changes. The median tumour volume reduction in tumours with a pathological response was 78.3% (range=16.5–97.5%) and for the no-change category was 67.5% (range=6.4–94.4%), the difference between the groups was non-significant (*P*=0.59 by Wilcoxon rank test).

### Marker immunohistochemistry

#### Mib1 staining

**Figure 1:**
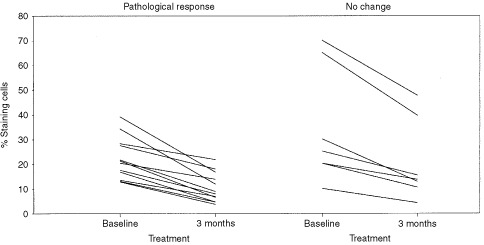
Changes in Mib1 (%) after 12 weeks of treatment with anastrozole (combination of 1 and 10 mg doses) in tumours with a pathological response to treatment (left panel) and in tumours with no response to treatment (right panel).

Measurements of Mib1 expression showed that treatment with anastrozole was associated with a reduction in staining score in all cases (Figure 1

). The initial levels and degree of reduction was not significantly different in tumours with and without pathological response.

#### TUNEL

**Figure 2:**
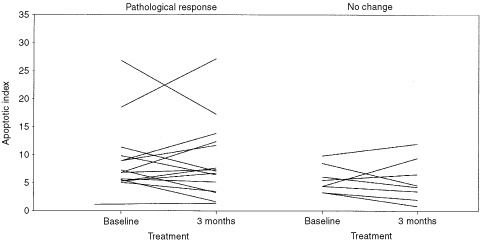
Changes in apoptotic index (‰) after 12 weeks of treatment with anastrozole (combination of 1 and 10 mg doses) in tumours with a pathological response to treatment (left panel) and in tumours with no response to treatment (right panel).

Changes in apoptotic index were noted with treatment (Figure 2

). However, the direction of change was not consistent, decreasing in 12 tumours and increasing in 11 tumours. Furthermore, pattern of change was not related to pathological response.

#### Oestrogen and progesterone receptors

In terms of effects on ER expression, treatment was associated with no change in the intensity and proportion of cells staining in 15 cases; in the remaining eight tumours there were minor changes in category score for staining parameters, but these were minor and equally increased or decreased.

Of the 23 tumours, 18 were assessed as being PgR positive before treatment (Table 3

**Table 3:**
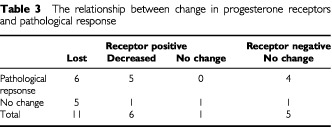
The relationship between change in progesterone receptors and pathological response

), scoring between category 3 and 8; of these, 11 (61%) subsequently displayed a pathological response. Of the five PgR-negative tumours, four (80%) showed evidence of pathological response and none changed their PgR status. However, anastrozole treatment caused a marked reduction in expression of PgR in 17 of the 18 receptor-positive tumours (in 11 cases this was a total loss). This reduction in expression was irrespective of pathological response.

## DISCUSSION

The results of this study show that neoadjuvant treatment with anastrozole may have marked effects on histopathological features within breast tumours. These include changes in histological grading features, markers of proliferation and cell death, and of PgR expression. We believe that this range of observation has never been previously reported for an aromatase inhibitor. It is therefore important to discuss these findings in terms of: (1) their relationship to clinical response, (2) their inter-relationships, and (3) a comparison with the effects of other endocrine agents, most notably tamoxifen.

Clear pathological responses in terms of reduced cellularity and/or increased fibrosis were noted in the majority of tumours (15 of 23; 65%). This high response rate probably reflects the patient selection criterion based on ER-rich tumours. It is, however, lower than the clinical response frequency in the same tumours. Furthermore, pathological response only correlated poorly with clinical response as determined by ultrasound. Thus, although the majority of tumours that had a substantial volume reduction with treatment also showed a pathological response (13 of 18), a minority had an ultrasound response without evidence of pathological changes. This is perhaps not surprising since it is possible to visualize a scenario in which tumours shrink without altering their microscopic morphology. Conversely, most tumours failing to show a significant reduction in tumour volume were also without evidence of pathological change. Furthermore, there were two tumours that did not have a significant reduction in tumour volume by ultrasound but yet had a pathological response. In these particular cases it may be that ultrasound measurements (which in our hands generally appear to give the most accurate assessment of tumour burden (Forouhi *et al*, 1994) did not accurately reflect true response. Indeed, calliper assessments in these particular cases indicated reductions in tumour size (54 and 99%) which were more compatible with the small pathological size of the excised lesion (data not shown). These observations suggest that, in monitoring the response to endocrine agents, pathological determinants should ideally supplement more traditional clinical tumour measurements.

In addition to changes in cellularity, other pathological features were altered with treatment in approximately 50% of cases: these included tubule formation, decreased nuclear pleomorphism, and decreased mitosis, which occurred both alone or in combination. This indicates that anastrozole therapy is capable of modulating cellular populations within individual tumours. Furthermore, these changes are generally towards a phenotype that is accepted as being less aggressive.

The effects of treatment with anastrozole on Mib1 expression were striking and consistent in that the proportion of staining cells was always less in the treated tumour as compared with the pre-treatment biopsy. Whilst the degree of reduction varied widely between different tumour pairs, this was not related to pathological changes or degree of tumour shrinkage, ER level or PgR status. It would therefore seem that one of the more important mechanisms of action of anastrozole, at least in its chronic use, is to take tumour cells out of the cycle of division. However, whether this then translates into a clinical or pathological response depends on other factors. The practical implication of these observations is that measurement of Mib1 staining, either initially or after 3 months' treatment, is not helpful in predicting/monitoring response. Whether intermediate time-point measurements would have had more utility is unresolved, but is worthy of further study. It is interesting to note that whilst we and others have observed consistent decreases in proliferation following therapy with aromatase inhibitors (Geisler *et al*, 2001; Harper-Wynne *et al*, 2002), this was not our experience with tamoxifen (Keen *et al*, 1997). Tamoxifen treatment could be associated with an increase in proliferation markers, particularly in non-responding tumours. Effects on such markers may be representative of an important difference in the mechanism of action between tamoxifen and aromatase inhibitors.

Changes in apoptotic index were observed with treatment. However, the direction of change was not consistent and did not relate to clinical or pathological response or change in Mib1 score. Assessment of these changes in apoptosis is complicated by the event being: (1) transient and of low frequency in most breast cancers, and (2) the result of either a primary response to treatment (successful therapy would increase score) or being secondary to changes in proliferation (decreased proliferation would produce a fall in score). Given that the chronology of the processes of proliferation and apoptosis may differ in individual tumours, the assessment of apoptosis at a single time-point of 3 months into treatment is almost certainly suboptimal. Given that, in experimental models, the primary apoptotic response to endocrine therapy precedes that of proliferation and may be seen as early as 2–3 days (Cameron *et al*, 2000), future studies of neoadjuvant anastrozole would benefit from the inclusion of tumour samples early in the treatment protocol. These studies are currently underway.

The lack of a consistent effect on ER expression observed in this study is also in agreement with results reported previously with other aromatase inhibitors (Sasano *et al*, 1999; Harper-Wynne *et al*, 2002). This contrasts with influences on the PgR, the expression of which was reduced in all but one case and was totally lost with treatment in most cases. The results of the present study are similar to previously reported data on other aromatase inhibitors (Sasano *et al*, 1999; Harper-Wynne *et al*, 2002) and are compatible with the PgR being an oestrogen-inducible protein. It should be noted that the decrease in PgR occurred in both tumours, with and without clinical or pathological response. Lack of clinical or pathological response is therefore not because the tumour fails to recognize anastrozole as an oestrogen-depriving therapy. It should be noted that in this setting, PgR status in the pre-treatment biopsy was not predictive of clinical or pathological responses: for example, four of the five PgR-negative tumours exhibited clear pathological responses with treatment. The consistent effect of anastrozole in decreasing PgR staining is further evidence that the drug's mode of action differs from that of tamoxifen, which has variable effects on PgR, including increased expression (Miller, 1996; Miller *et al*, 1999; Miller *et al*, 1999; Chang *et al*, 2000). The difference in phenotypic expression following treatment with tamoxifen and aromatase inhibitors may have clinical relevance in terms of resistance to treatment and the choice of subsequent sequence of therapies (Hu *et al*, 1993; Macgregor and Jordan, 1998). Interestingly, aromatase inhibitors have already been shown to be of therapeutic value for many patients whose tumours have developed resistance to tamoxifen (Bajetta *et al*, 1999).

In summary, the neoadjuvant use of anastrozole has been shown to have clear effects on histological features of breast tumours. The pathological findings that anastrozole decreases the expression of both PgR and Mib1 are particularly striking, and reflect the powerful anti-oestrogenic and anti-proliferative potential of the drug. However, the present study should be regarded as a pilot study, and the intermediary mechanisms by which anastrozole achieves a clinical or pathological response remain unclear. Further research in this area should include a larger study population, and samples taken earlier after the start of treatment.
